# Trust, negotiation, and communication: young adults’ experiences of primary care services

**DOI:** 10.1186/1471-2296-14-202

**Published:** 2013-12-30

**Authors:** Antoinette Davey, Anthea Asprey, Mary Carter, John L Campbell

**Affiliations:** 1 Primary Care Research Group, University of Exeter Medical School, St Lukes Campus, Smeall Building, Magdalen Road, Exeter EX1 2 LU, UK

**Keywords:** Young adults, Primary care, Communication, Trust, Negotiation, Satisfaction

## Abstract

**Background:**

Young adulthood is an important transitional period during which there is a higher risk of individuals engaging in behaviours which could have a lasting impact on their health. Research has shown that young adults are the lowest responders to surveys about healthcare experiences and are also the least satisfied with the care they receive. However, the factors contributing to this reduced satisfaction are not clear. The focus of our research was to explore the needs and experiences of young adults around healthcare services with an aim of finding out possible reasons for lower satisfaction.

**Methods:**

Twenty young adults were interviewed at GP surgeries and at a local young adult advice agency, exploring their experiences and use of primary care services. Interviews were analysed using thematic analysis.

**Results:**

The use of primary care services varied amongst the young adult interviewees. Many interviewees reported positive experiences; those who did not linked their negative experiences to difficulties in negotiating their care with the health care system, and reported issues with trust, and communication difficulties. Most of the interviewees were unaware of the use of patient surveys to inform healthcare planning and delivery and were not inclined to take part, mainly because of the length of surveys and lack of interest in the topic area.

**Conclusions:**

In order to effectively address the health needs of young adults, young adults need to be educated about their rights as patients, and how to most efficiently use primary care services. GPs should be alert to effective means of approaching and handling the healthcare needs of young adults. A flexible, varied approach is needed to gathering high quality data from this group in order to provide services with information on the changes necessary for making primary care services more accessible for young adults.

## Background

Patient experience of NHS services has been at the forefront of recent political and healthcare agendae. Experiences and perceptions of primary care services have been documented nationally via the GP Patient Survey (GPPS) since 2006/7. This survey provides healthcare professionals in England and Wales with information regarding access, health professional communication styles, and overall patient satisfaction. Young adults’ response rate to the GPPS, however, has been consistently the lowest compared to other age groups and reasons for this low response rate are unclear [1]. Additional national and local surveys are also used by primary and secondary care services to gather data on patient experience [2-6]. Although national surveys gather and report experiences of young adult patient across the country, many of the questionnaires have been piloted and cognitively tested with patients aged 18 and over. This brings to question the reliability of results from these surveys around young adult experiences when the specific wording and comprehension of questions have not been tested for 16 to 18 year olds. Results from such surveys provide policy makers and practitioners with an overview of service performance and identify areas where improvements may be made in order to more fully meet patients’ health needs.

Children and young people (under the age of 24) constitute at least 40% of GP workload, but levels of satisfaction with primary care services for young adults (18 to 24 years) remain lower in comparison to older adults [1,7,8]. A small number of qualitative studies and consultations reporting children and young people’s experiences of primary and secondary care reveal that poor communication, less involvement in decisions about their care, and an unfriendly environment leads to dissatisfaction with services [7]. However, these studies have focused on both children and young people, with less emphasis on young adults as a separate age group. A major national UK report suggested that these lower levels of satisfaction may be a consequence of children and young people’s services not being prioritised with regard to the management and delivery of services, and allocation of funds [9]. A lack of current investment in the health care needs of a young adult population may increase the need for such resources in the future.

Although there are substantial international data on patient experience of health care services, there is a lack of focus on healthcare experiences of young adults. This is despite growing recognition of the increasing size and importance of this population group [10], and the need for better understanding of their health status [7,11] and specific healthcare needs [12]. Studies conducted in the US revealed that young adults experienced difficulties when accessing primary care services, and highlighted the importance of this transitional period in a young person’s life [13-16]. In the UK, the Kennedy Report specifically recognised the importance of young adults as a separate population from children and adults, needing specific awareness among health professionals regarding the delivery of healthcare.

Although young adults are generally thought of as a healthy population [17], recent studies in North America [16], Europe [18] and Canada [19] reveal a different picture. Significant changes in the timing and sequencing of the transitions to adulthood (e.g. later age at first marriage, birth of first child, leaving the parental home, becoming economically independent and leaving school) have impacted on the health status of this age group [19]. Young adulthood is a key time of transition, during which individuals may engage in health risk behaviours [20] such as eating disorders [21], risky sexual activity (such as unprotected sex) [13] and substance abuse [19]. It is reported that the leading cause of mortality in the United States for young adults is linked to road traffic accidents (RTA) [22], and although in the UK RTAs account for less than 1% of all deaths, the highest incidence of mortality is amongst the young adult age group [23]. Adverse physical and psychosocial health-related behaviours during young adulthood could potentially impact on future health status, healthcare needs [18], and use of services by this age group.

Due to the peak in risk taking behaviours during young adulthood [16], research suggests that young adults may fare worse on national health indicators when compared with other age groups. Despite this, there has been little attention paid to developing a national strategic plan that addresses the needs of this population group [15]. Similarly in the UK, research and service design has tended to focus either on children and adolescent services or on adult services. The new NHS Outcomes Framework [24] highlights five key domains with specific indicators designed to measure the outcomes of effectiveness of care, quality of patient experience and patient safety. An additional indicator, specifically aimed at addressing the healthcare experiences of children and young people, was recently included under the fourth domain “Ensuring that people have a positive experience of care” [24].

The age definition of ‘young adult’ varies across agencies, with some defining a young person as aged between 16 and 24 years [25], whilst others define those aged between 18 and 21 years as young adults [26]. For the purposes of our research, we have defined young adults as anyone aged between 18 and 25. The aim of this study was to explore young adults’ needs and experiences of primary healthcare with a view to identifying the reasons for lower satisfaction with regard to services, in addition to understanding why young adults are less likely to respond to patient experience questionnaires.

## Methods

### Setting and participants

The study was conducted in two settings in Devon, UK - in 4 GP practices, and in a local advice agency targeting the needs of young people (Connexions). The GP practices differed in terms of list sizes, location and socio-demographic profile. Patients attending appointments were approached in the waiting room and provided with a brief background to the study and invited to take part in an interview regarding their experiences of using primary care services, their experience of and attitude towards patient surveys.

Participants were approached and provided with a recruitment pack (which contained an information sheet, response form and prepaid envelope) and asked if they were able to spare some time to take part in an interview at the point of recruitment. If this was not possible a suitable time and date was arranged to carry out the interviews either at the GP practice or the patient’s home.

Connexions, provided permission to recruit and to conduct interviews in one of the private offices on the premises. At the time of this study, Connexions provided an information, advice, guidance and support service for young people aged 13 to 25, around education, employment, housing and healthcare. Young adults were provided with the same information about the study as those recruited in GP practices.

Twenty young adults agreed to take part in face-to-face interviews in either the GP surgery (*n = 4*) or at the local young adult advice agency (*n = 16*). Initial attempts to recruit young adults from GP surgeries were not very successful, with a total of 10 visits covering both morning and afternoon surgeries at four GP surgeries. A total of 52 invitation packs were distributed and six young adults agreed to take part; two subsequently withdrew from the study. Over the course of 2 visits to Connexions a total of 25 young adults were invited to take part, nine declined and 16 young adults were interviewed. Heterogeneity was sought by purposively sampling equal numbers of participants from a younger age group (18–21 years) and older age group (22–25 years), as well as a sample of each gender.

### Data collection

Semi-structured interviews were conducted by AD using a topic guide which was developed following a literature review [27] identifying the views and priorities of young adults. The questions covered young adults’ use and perception of primary care services in general, their health needs, expectations regarding health professionals and their experience of surveys. Each interview was audio-recorded with the participant’s consent and interviews lasted between 30 and 60 minutes.

### Data analysis

All interviews were transcribed verbatim and then organised for analysis in QSR’s NVivo 9(28) software by two researchers (AA & AD). Data was analysed using thematic content analysis. The transcripts were read and were coded according to themes. Themes were modified as further data was collected and analysed. Identification of the themes came from the data and influenced by a literature review conducted prior to the study. A third researcher (MC) blind coded a random selection of 3 transcripts in order to ensure accuracy and consistency across our analyses. Ethical approval for the study was granted by Devon and Torbay NHS Research Ethics Committee.

## Results

Over half of the participants were female (65%) and the average age across the sample was 20.75 years. Ninety-five percent of the sample were White British, and only one young adult was from an Asian ethnic group. These figures reflect the population profile of the area, with over ninety percent being from a White British ethnic group [28]. Over half the young adult males were currently employed (*n = 4*), one was studying at the local tertiary college, and two were unemployed. Of the females, five were studying at the local college, four were working and four were unemployed. In the city where most of the interviews took place there are two Walk-In Centres, one in the city centre and one on the hospital site, located at about one mile from the city centre.

### Overview of registration at GP practices

Although most of the young people were registered with a local GP, some (n = 3) were not currently registered. Lack of registration was connected with some kind of residential impermanence, such as having recently moved into the area. Frequency of attendance varied greatly from several times a month to only once in four years. Half of the interviewees (n = 9) had used Walk-In Centres, either for contraception, other sexual health matters, or when they could not get a quick appointment at their GP surgery, such as for injuries.

### Emerging issues

There were issues emerging from the data suggesting that specific problems are encountered by this particular age group. These were:

Difficulties with negotiating the primary care system

Issues with mutual trust

Communication difficulties

### Difficulties with negotiating the primary care system

As young people in this age group are undergoing the transition from dependence upon their parents to an independent adult life, the task of negotiating their way around the primary care system was often new to them. This inexperience may explain the tendency for some young people in the sample to have a preference to attend walk-in centres, which allows them to avoid arranging an appointment with their GP surgery:

*“I just don’t like making appointments, not always very good at remembering stuff, so I’d rather just sort of walk in”.* (603 Male aged 22 years)

More than half of the young people said they had used a Walk-In Centre, and although this was sometimes for the same reasons as any patient might have, such as needing to see a doctor out of surgery hours or because the location of the centre was more convenient, other reasons given were more likely to be specific to the young age group (see Mutual Trust and Communication sections below).

Some young people had found difficulties with navigating the appointment system in general practices and believed that it was impossible to get quick appointments, even when a medical problem was serious:

*“I have tried to ring up and get an appointment on the day and they couldn’t until a few days later … but what if I had a pretty serious problem or something or something was going on and you couldn’t see me today, what was I supposed to do?”*. (607 Male aged 18 years)

However, some young adults were aware of the way the appointments system operated which appeared to be acceptable and suitable to them:

*“If you ring up before 10 in the morning they well get almost like a duty doctor to phone you back … and they will decide if you need to be seen that day or within three days, so it’s very, very quick there”.* (611 Female aged 19 years)

There was further evidence of a lack of familiarity with the service available in doctors’ surgeries. For example, one interviewee was unfamiliar with patients’ rights and thought it was not possible to choose which doctor he consulted:

*“Yeah, I would* [prefer to see the same doctor], *but I didn’t think I had the choice or, you know, a say in it, just would see who was there”.* (607 Male aged 18 years)

The sometimes unstructured nature of some young adults’ lives could also act as a barrier to effective use of general practice services, particularly when there was little flexibility in the system. This young woman, for example, found it hard to organise herself to make appointments at the appropriate time:

*“You got to book an appointment, you got to rung them up about 8.30 or 9 a.m. to get an appointment but if you don’t then you got to ring up the next day … It’s weird, honestly, ‘cause I went there and said ‘Can I book an appointment for tomorrow, and they say you got to ring up tomorrow, we can’t do it ….if like you wake up late you can’t get an appointment, you’re already stuck … it’s ridiculous, ‘cause if you got no credit on your phone or … if you wake up late …”* (619 Female aged 24 years)

Although some young adults appeared to be less knowledgeable or experienced in negotiating the appointment system, some individuals were extremely able to negotiate different aspects of the service:

*“I just phone up and just say is there any chance I can be seen today and I’d say to them a little bit of what it’s about … then they’d book me an appointment … If I phone first thing in the morning, then I can get in on the same day, if not then I’d have to either come in a couple of days later or the following week but then sometimes if I go in the following week I’m feeling fine so I don’t have to go in but most of the time I get in on the same day”. (*605 Female aged 18 years)

### Issues with mutual trust

The young people were asked what was important to them when they attend their GP surgery. A key issue that emerged in response to this was the question of trust between the doctor and the patient. It was clear, for example, that for some of the young people the ideal primary care provider was their own GP, where trust had been built over time through continuity of care:

*“I think it’s that like confidence in him … I know him as my doctor, than just going into the walk-in* [centre] *and seeing someone who doesn’t know anything about me … he would know what was right for me”.* (609 Female aged 18 years)

In addition, familiarity with the doctor appeared to have an impact on the ease with which they could talk to him or her and confidence that they can give the doctor the relevant information:

“*I would rather speak to the same GP, ‘cause then they know what kind of person you are and everything like that and you feel a bit more confident, I think”.* (613 Female aged 17 years)

*“It’s good to have confidence in the doctor that you have because you might have an embarrassing problem and if you don’t feel comfortable around seeing a new doctor … it can be quite hard to open up to him … if you’ve had someone else who you’ve had for a while it’s easier to tell them”.* (607 Male aged 18 years)

Most of the young people appreciated being able to have appointments with their own GP and stressed the importance of continuity:

*“I just think it’s better to have a relationship with one than keep seeing another ‘cause then you know … he or she won’t only be your doctor also be your friend as well … I’d like to stick to just one person and them know my history and … someone to connect to as well”.* (607 Male aged 18 years)

It was important to many interviewees that the doctor should put them at ease, listen to them and take them seriously:

*“When I talk to my doctor she is actually listening to me and understands what I mean”. (*605 Female aged 18 years)

*“To feel comfortable really. Yeah, to feel like you are not being kind of intimidated or whatever um and just feel like you can you know say what you’d say and not feel uneasy or whatever”.* (613 Female aged 17 years)

Others were concerned that their GP should be professionally competent:

*“I would want to be reassured that the doctor was competent and knew what he or she was doing”.* (602 aged Male 22 years)

Despite the high importance of these matters to the young people a number of interviewees gave accounts of incidents or situations which indicated a breakdown in trust. When they were unable to see their own doctor and had appointments with locums or other GPs, some young people felt this had undermined accurate diagnosis:

*“When he* [my own GP] *is like not there, I’ve seen the odd doctor … but then she told me to come back to him, because like she wasn’t sure”.* (609 Female, aged 18)

*“When I’ve been before they* [the locum] *couldn’t really tell what was wrong with me, ‘cause my doctor was ill* [and could not see her]*”.* (605 Female aged 18 years)

Some interviewees reported encounters with their GP where they had felt they had not been taken seriously and therefore not accurately diagnosed. One young woman recalled a time when she had approached her GP with a health concern but was ignored when she requested an investigation. According to her, this had resulted in a collapse and hospitalisation:

*“It took them six months to actually do something and it’s only ‘cause I collapsed at school … I am a bit nervous of doctors, ‘cause they’re not too good down there”.* (611 Female aged 19 years)

Subsequently, she was relieved when her present doctor picked up on the condition that previous doctors had dismissed. She was also upset that, in her previous surgery, her concerns about her younger brother were not taken seriously:

*“There was an incident with my brother, um…he come down with a rash all over his body. I panicked so I took him to the doctor’s and they just go ‘Oh, it’s just a virus’, two days later he was rushed to A&E with measles”.* (611 Female aged 19 years)

In another case a young woman felt the doctor was not trusting her to tell the truth and undertook a blood test without her fully informed consent:

*“Once, I went in and I was getting some bleeding between periods, and it was worrying me and she basically just kept on asking me if I had Chlamydia and I said I knew I didn’t, and I said no a lot of times and she kept on going on about it, and then said ‘Okay we’ll do some routine tests’ and actually she just did a Chlamydia test, which is what I saw on the screen [laughs]”.* (604 Female aged 22 years)

Another breakdown in trust that was mentioned by the young people related to concerns regarding breaches or potential breaches of confidentiality. One interviewee had experienced this when the GP talked to her aunt (with whom the young woman was currently residing) about something the young woman had shared in a consultation:

*“My auntie managed to ring up, had a discussion with my GP. My GP then spoke to her about information I’d spoke to her* [about] *in my appointment, so of course as soon as I got home my aunt was yelling at me, I was like ‘where did you get that from?’ She was like ‘I was speaking to your GP’, I was like ‘I’m 18, you shouldn’t be able to speak to my GP’. I was really not happy that she had managed to have a conversation with her on the phone, so I just said ‘Right, I’m not seeing her, I am going to someone else”.* (611 Female aged 19 years)

Other young women in the sample expressed concerns about the level of confidentiality offered by primary care services. There was a particular concern that the family GP would not respect their need for this with regard to contraception services:

*“For the contraception pill and stuff … I think I didn’t go to start with* [to the GP] *because I thought my mum would find out at the beginning”.* (608 Female aged 18 years)

*“I don’t really go to my own doctor that much because … my mum goes there as well and I don’t want anything that I say to my doctor goes to my mum … that’s another reason that I go to the walk-in centre”.* (605 Female aged 18 years)

Indeed, one reason Walk-In Centres were used by both young women and men was for matters related to sexual health. One young man, for example, who explained that despite the fact that he had a long and close relationship with his own GP, *“I’ve been with him since, well, a long time ago … he knows exactly what’s going on”,* he nevertheless chose to use the Walk-In Centre for what he euphemistically called *‘other problems’* (606 aged 19 years). Although he did not connect this behaviour directly with the issue of confidentiality this may have played a part in his decision.

### Communication difficulties

As mentioned above (Issues with mutual trust) the interviewees stressed the need for good communication with their doctor, who ideally would listen to them and explain things clearly and easily.

Sometimes when the young person was unable to see their own doctor, there appeared to be a breakdown in communication, which could lead subsequently to a reluctance to go for treatment:

*“I had a bad experience the other day where I went because I’ve got chronic fatigue syndrome and my university wanted a letter that generally just explained the symptoms … and I … had an appointment with* [name of own GP] *but he was ill, so I had a locum doctor and he just basically refused to give me one … which I thought was a bit out of order. I kept explaining the situation but he just didn’t seem to quite understand … the only thing that puts me off is that if* [name of own GP] *is not there then I don’t really want to go”.* (604 Female aged 22 years)

Another issue that was raised by the young people was the kind of language that doctors use in consultations. Some experienced this as a barrier to understanding, especially when specialist medical language was used:

*“If they say something is wrong and they use all these long words, for me that is confusing, I sit there and go ‘what?’ and just nod my head and agree when actually I have no idea what they are saying. so if I could change that I would have someone, I’d have the doctor explain it in more of a simpler form”.* (618 Female aged 18 years)

It was also suggested that the level of communication in respect of practice services might sometimes be lacking, resulting in young people being unaware of what services were offered there, and so going elsewhere for help. One young woman (611) who seemed particularly independent, having moved on her own to the area from a considerable distance away at the age of 17 years, commented that many of her friends were unaware that they could go to their GP for contraceptive services or other sexual health issues:

*“It’s not advertised at all, especially in my surgery, I don’t see nothing that says, you know, contraception advice, come here … I’ve got friends who will go to the Walk-In Centre, but I’m like ‘You can go to your GP as well’ and they’re like ‘Oh, can you?’ … ‘cause I’ve got a friend at the moment who thinks she might be pregnant, and I’m like ‘Go see your GP’ and she’s like ‘Oh, but then he’ll find out’ I says ‘Well, if you’re pregnant he’s going to find out anyway!’.”.”* (611 Female 19 years)

However, some interviewees expressed positive experiences with the way their GPs communicated with them. They were appreciative when their GP was sympathetic, listened to them and gave them time and the opportunity to explain themselves:

*“The doctor was really helpful with what I needed, anyway, I mean, I just went there for a chat really, I wanted to sort out my head a bit … so yeah, I had a good chat with him, asked him about a few things, and just very helpful”.* (603, Male aged 22 years)

*“I think she’s lovely, I’ve had her for many years so … the fact that when I talk to my doctor she is actually listening to me and that she understands what I mean …”* (605 Female aged 18 years)

Interviewees also made positive comments when their doctor took time to explain things to them clearly and made appropriate referrals:

*“The doctor I saw … was really, really good, um, he actually went into quite a lot of detail … gave me information … also referred me to a counsellor and everything as well, so he was really like in-depth and helpful about it”.* (601 Female, aged 25 years)

### Young adults and patient surveys

Interviewees were also asked for their views about patient feedback surveys, whether they had taken part in them or whether they thought they would do so if they had the opportunity. They were also asked how best such information might be gathered from young adults and whether they would consult the resulting data to inform them about their doctor’s surgery. Only one interviewee was sure that she had taken part in a patient survey and one other remembered being sent one, but did not participate. Others were mostly completely unaware of such surveys.

Two of the interviewees took the idea of patient surveys seriously and wanted the opportunity to feedback their experiences and views:

*“Obviously that’s going to be our future anyway … I think it’s important to us and I think we are pushed aside a little bit because we are the younger generation… we are just as much of a priority as [older] people”.* (613 Female aged 17 years)

*“I think it’s important that every one that goes to the doctors has a say in how they think the surgery is and how the doctors are. I think it’s important that they know how the public feel about the service they get”.* (616 Female aged 22 years)

A number of the interviewees, however, felt that the majority of their age group would not be interested because of the lack of interest in the topic area:

“*Really it’s just about that age range’s unwillingness to do anything at all …I think that might be something you will be struggling with these questionnaires, because young people aren’t particularly interested in their health”.* (602 Male aged 22 years)

*“I’d probably do it but, speaking on quite a lot of people’s behalf I … think they will just throw it away straight away”.* (617 Male aged 22 years)

Attitudes toward patient surveys varied but were mostly quite ambivalent. For example, the one young person who had taken part in a primary care survey admitted that she had only done so because her parent suggested it was useful, “*but truthfully if I hadn’t had the external pressures then I probably wouldn’t have bothered to do the whole thing”* (604 Female aged 22 years).

Around a third of the interviewees said they would be unlikely to participate in a patient survey and the rest stated that, hypothetically, they might be interested, with various caveats. For example, some said they would respond if the survey was short and restricted to tick boxes, some said they would only be likely to complete one if it was online and an incentive was given. Others felt they would be most likely to respond if it was presented in the waiting room of their surgery to be completed in situ.

Given the proliferation of social networking amongst young people it was thought that perhaps online surveys might appeal more to the interviewees, but some uncertainty was expressed, and it was felt that an incentive would be needed:

*“I don’t think they would be interested, um, I think if it was important … the best way would be like internet or something, but then they would need an incentive to actually go on and do it”.* (601 Female aged 25 years)

*“Facebook’s quite a good idea but whether people would actually sort of pay attention to it or not … suppose a lot of people just sit on Facebook doing nothing really, so you might attract those sorts of people”.* (603 Male aged 22 years)

Others felt that a questionnaire presented to young people in their doctor’s surgery would elicit a better response than a postal survey

*“I think may be while you are in the surgery, because if you get it sent to your home you are less likely to do it if it’s sat on the kitchen table, whereas if you go into the surgery and you are waiting to see the doctor you can do it then”.* (616 Female aged 22 years)

One interviewee thought the best place to distribute surveys was in college environments where they could be structured into the timetable:

*“They [the college] will whack this survey in as part of the course, so that the students will have to do it at the start of the course … I saw loads of random surveys that have nothing to do with the college when I was at college … it was annoying but we had to do it..”* (615 Male aged 23 years)

Some of the interviewees said they were interested in viewing the results of their own GP surgery’s patient survey, although it was generally felt that results available in the surgery waiting room were preferable to online information. Others, however, felt that personal experience was the best gauge of quality of care, *“if my doctor is nice and they don’t make me ill, then I think it’s fine”* (608 Female aged 18 years).

## Discussion

In this sample, young adults’ use of primary care services varied from rare attendance to frequent visits. Reported experiences of primary care services and with professionals were generally positive. However, the transition from adolescence to adulthood appears to give rise to specific problems for young adult users, including difficulties negotiating the primary care system, issues with mutual trust and communication difficulties with their GP. An accumulation of a lack of experience in negotiating and understanding the healthcare system and a problematic relationship with the GP led to some young adults using other, more obviously available primary care services, such as the walk-in centre.

The idea that young people lack the cognitive or psychosocial maturity to effectively use the health care system is not novel [29], but it is apparent that difficulties continue to be experienced. Young adults are at a stage in their lives where they are developing an increased autonomy in every aspect of their life, including the decision to seek medical advice when needed. Some young adults lack understanding of how the health care system works, what their rights are as a patient, and what services are available to them from their local GP surgery. In addition to this, the ever changing landscape of primary care can be challenging to keep up to date with for young adults. For example, many practices have embarked on changing appointment systems to a total triage model which may either hinder or make access to services easier for this age group. However, effects of these changes on access and care for young adults have not been researched or explored. As shown in this study, young adults’ inexperience and lack of knowledge of current primary care systems can lead them to use other more accessible services such as a walk-in centre or the accident and emergency service within a hospital, impacting on their workloads.

There is recognition that a smooth transition from child and adolescent care to adult care for individuals suffering from chronic physical and medical conditions is important and best practice guidelines have been developed to ensure services address the needs of young adults during this transitional period [30,31]. These guidelines do not, however, extend to the general young adult population not suffering from any chronic physical or medical condition. As young adults become responsible for their healthcare during this transition, there is an increasing need to support them in developing their communication skills, assertiveness and decision making skills in order to effectively manage their own healthcare.

Effective communication between the patient and GP is integral in any successful consultation. Our research has shown that young people consider the communication skills of both the healthcare professional and the patient to be important aspects of the consultation process. There are difficulties in this regard as our results showed that some GPs are not perceived to take young adults’ health concerns seriously or to explain things clearly. The Royal College of General Practitioners (RCGP) recognise the importance of GP communication and how effective communication skills can help build trust in the GP-patient relationship which can be strengthened over time through continuity of care [32]. This formula of effective communication, trust and continuity of care can improve patient reported health outcomes and patient satisfaction. Some interviewees in this study reported problems conveying their symptoms and being adequately listened to by their GP; this could potentially lead to an inaccurate diagnosis and an inappropriate treatment plan. In such circumstances a lack of trust may then develop regarding the GP’s capabilities and the service they provide, which can cause young people to stop using that particular service and seek alternative care. As mentioned previously, there is a tendency among young adults to use alternative services such as the walk-in centre or A&E can create an unnecessary, additional strain on those services. Recent data published by the Health and Social Care Information Centre report that the 18–28 age group represent 18% of attendances at all A&E units and walk in centres and minor injury units see a higher proportion of younger patients in comparison to major A&E units [33].

The introduction of the salaried service for GPs where the length of the contract may be shorter also means that GPs can move practice more often. Providing stability and continuity of care with familiar health professionals, which has emerged as important to young adults, will be a challenge to the new generation of primary care providers who are more mobile than their predecessors. Understanding how GPs move between different practices has an effect on the provision of continuity of care to patients is important as it has been identified by young adults as a significant aspect of care, however it is currently under-researched.

Another concern raised by interviewees related to informed consent and confidentiality, especially with regard to sexual health issues. There was also a lack of understanding about their rights as patients once young people become legally responsible for their own healthcare. Our findings suggest that specific targeted leaflets providing young adults with information regarding their rights as a patient may be beneficial in educating, empowering and enabling them to take responsibility for their healthcare. Assurance from the GP about the confidential way in which personal information is handled is essential early on in the process, as this will assist in the development of mutual trust between the patient and GP.

Communication problems are also experienced at a practice level as young adults are unclear about the services that are offered within their own surgeries. The lack of clarity around service availability lies both with the clinician and patient. Due to technological advancements and the increased use of social media, it is important for providers to consider creative ways to convey information about their services to the younger population. Equally, younger patients should be able to identify a preferred method for receiving information regarding their GP surgery. Our findings suggest that patients and providers should work together in order to achieve a service which addresses patient needs through the means of communication appropriate for the different age groups (e.g. use of email/text for younger patients and more traditional methods for older patients).

Every year the lowest responders to the national GPPS are young adults aged between 18 and 24. The attitudes of our young adult interviewees towards surveys were mostly ambivalent. The majority were unaware of the existence of a national survey, and only a minority of respondents stated that they would complete it. The reasons suggested for the low participation rates of young adults mainly related to the length of the questionnaire and the topic area. Many young adults expressed little interest in completing a long survey about their perceptions and experiences of a health care service. Despite this, some young adults recognised that their responses were important, as they might affect the services provided in the future. Incentives and online surveys were highlighted as possible methods of increasing response rates to such surveys, as well as shortening the length.

### Strengths and limitations

Despite previous quantitative studies investigating the perceptions and experiences of patients accessing primary care services, to the best of our knowledge, this is one of the few studies in the UK specifically focusing on the primary healthcare needs and experiences of the young adult population. Previous quantitative studies have shown that young adults report more negative experiences with their healthcare providers in comparison to other age groups [34,35], although reasons for this could not be explored. The data obtained from these interviews have contributed to filling the gap in knowledge about young adults’ perceptions of primary care and possible causes for lower rates of satisfaction with current services. Participants were from a range of ages and occupations, and reported a varied use of primary care services. Although the participants in this study were representative of the local population in terms of demographics, the sample does lack representation from ethnic minority and NEETs (not in employment, education or training) population groups. The views of young adults from these groups need to be explored in order to enrich our understanding of experiences of young adults in general.

Another limitation of this study was the lack of sufficient information about non-responders; we were unable to assess whether or not they had different characteristics from those who participated. The demographic details relating to our participants were diverse, however, and there was a wide range of experiences reported.

## Conclusions

The World Health Organisation has identified young people’s health as a global priority. Research conducted in England has shown that young adults report the poorest experience in health care in comparison to all other adult age groups. However, this is not limited to the UK and similar findings have been reported elsewhere resulting in young adults making the decision not to use that service again [36,37]. Change may be needed both in terms of educating young adults about how to use primary care services as they approach the transition from dependent child to independent adult, but GPs may also need to be informed about how best to approach this group of patients. It is globally recognised that there is a need for a more youth friendly service within primary care, however young adults are still reporting poor experiences when using primary care services. Various youth-friendly health initiatives have been developed across the world, research has shown that the benefits of these initiatives on the health of young people have not been appropriately documented [38].

Given the potential barriers to full use of primary care services, special attention needs to be paid to the manner in which data is gathered from young adults in patient surveys, so that good quality information is obtained about experiences and improvements subsequently implemented. Surveys completed in the surgery or in college environments may be the best way to obtain information, but a multi-method approach may well be successful, including online options (with the provision of incentives) and options exploring technological platforms (such as mobile phone surveys or in-practice real-time data collection). Failure to specifically address the health needs of young adults may represent a false economy, with inadequate addressing of health problems now stirring up future challenges not only for NHS healthcare, but for healthcare services across the world.

## Competing interests

The authors declare that they have no competing interests.

## Authors’ contributions

All authors contributed to the design of the study. AD conducted the interviews. AD and AA carried out a detailed analysis of the dataset. MC separately coded a selection of the transcripts. The various themes were discussed with all authors. AD and AA prepared the manuscript and all other authors provided critical comments on the manuscript.

## Pre-publication history

The pre-publication history for this paper can be accessed here:

http://www.biomedcentral.com/1471-2296/14/202/prepub
